# Detecting Human-to-Human Transmission of Avian Influenza A (H5N1)

**DOI:** 10.3201/eid1309.07-0111

**Published:** 2007-09

**Authors:** Yang Yang, M. Elizabeth Halloran, Jonathan D. Sugimoto, Ira M. Longini

**Affiliations:** * Fred Hutchinson Cancer Research Center, Seattle, Washington, USA; † University of Washington, Seattle, Washington, USA

**Keywords:** human influenza, outbreaks, surveillance, control, data analysis, mathematical model, research

## Abstract

Highly pathogenic avian influenza A (HPAI) subtype H5N1 has caused family case clusters, mostly in Southeast Asia, that could be due to human-to-human transmission. Should this virus, or another zoonotic influenza virus, gain the ability of sustained human-to-human transmission, an influenza pandemic could result. We used statistical methods to test whether observed clusters of HPAI (H5N1) illnesses in families in northern Sumatra, Indonesia, and eastern Turkey were due to human-to-human transmission. Given that human-to-human transmission occurs, we estimate the infection secondary attack rates (SARs) and the local basic reproductive number, *R_0_*. We find statistical evidence of human-to-human transmission (p = 0.009) in Sumatra but not in Turkey (p = 0.114). For Sumatra, the estimated household SAR was 29% (95% confidence interval [CI] 15%–51%). The estimated lower limit on the local *R_0_* was 1.14 (95% CI 0.61–2.14). Effective HPAI (H5N1) surveillance, containment response, and field evaluation are essential to monitor and contain potential pandemic strains.

## Abstract 2

Effective surveillance, containment response, and field evaluation are essential to contain potential pandemic strains

Highly pathogenic avian influenza A (HPAI) subtype H5N1 is repeatedly crossing the species barrier to humans. Since December 2003, a total of 291 cases of HPAI (H5N1) have been reported in humans, resulting in 172 deaths (i.e*.*, 59% case-fatality ratio) in 12 countries, mostly in Southeast Asia (*1*). Among these cases, 31 family clusters have been documented, ranging in size from 2 to 8 family members. How many of these clusters are due to a common avian source and how many are due to human-to-human transmission are important facts to determine. Should one of these HPAI (H5N1) strains gain the capacity for sustained human-to-human transmission, the resulting outbreak, if not contained, would spread worldwide through the global transportation network more rapidly than adequate supplies of vaccine matched to the new variant could be manufactured and distributed (*2*,*3*). We analyzed data from 2 of the largest of the familial clusters to ascertain if human-to-human transmission took place, and if so, how transmissible the strain was.

## Methods

### May 2006 Human Avian Influenza Family Cluster, Indonesia

During late April and early May 2006, a cluster of 8 cases of HPAI (H5N1) was detected and investigated by the Indonesian public health surveillance system in northern Sumatra (*4*–*6*). All case-patients were members of the same extended family. Seven of them resided within 3 adjacent houses in the village of Kubu Sembilang. The remaining patient resided with his immediate family in the village of Kabanjahe (≈10 km away).

The index patient was a 37-year-old woman, thought to have been exposed to dead poultry and chicken fecal material before onset of illness. She also reportedly maintained a market stall that sold live chickens. Although her illness was not confirmed to have been caused by the (H5N1) avian influenza virus, her death on May 5, 2006, is suspected to be the result of HPAI (H5N1) infection because of her reported symptoms, illness progression, and prior contact with diseased or dead poultry.

Twenty members of her extended family are suspected to have been in contact with her, many during a family gathering on April 29, 2006 (*7*). At that time, she was manifesting symptoms (i.e., she had a heavy cough, was severely ill, and was prostrate). That night, 9 of these members slept in the same small room as she did (indicated by a black triangle in Appendix Figure 1). Of these 9 family members, 2 of her sons (15 and 17 years of age) and her 25-year-old brother, who lived in Kabanjhe, became ill in the next 3 weeks. The sons died. The brother was the only person from this family cluster to recover.

Of the remaining 11 family members, 4 became ill and died. The 29-year-old sister of the index patient, who lived in an adjacent house, became ill after she provided direct personal care to her ill sister (*7*). The 18-month-old daughter of this sister also became ill after she was in the presence of the index patient with her mother. The 10-year-old nephew of the index patient, who lived in the other house adjacent to hers, became ill after he attended the family gathering and frequently visited his aunt’s house. The nephew’s father became ill after he personally cared for his son. The possibility that HPAI (H5N1) was transmitted from the nephew to his father is also supported by genetic sequencing data (*4*). Though symptoms did not develop in the mother of the nephew, she was directly exposed to her husband during his illness. All case-patients, except for the index patient, were confirmed as influenza (H5N1) positive by PCR. The nephew’s mother was confirmed as influenza (H5N1) negative. As an intervention, 54 surviving relatives and close contacts were identified and placed under voluntary quarantine (*7*). All of these persons, except for pregnant women and infants, received oseltamivir prophylactically.

### December 2005 Human Avian Influenza Family Cluster, Eastern Turkey

From December 18, 2005, (*8*) to January 15, 2006 (*9*), a cluster of 8 confirmed (H5N1) influenza cases was detected in Dogubayazit District in eastern Turkey (Appendix Figure 2) (*10*–*13*). These case-patients were among 21 members of 3 households located within 1.5 km of each other (*14*). All confirmed case-patients were hospitalized after onset of symptoms (*9*). Four of the confirmed case-patients died; the other 4 recovered (*9*). Ten of the remaining 14 household residents were hospitalized with avian influenza-like symptoms but were never confirmed to be (H5N1) infected (*9*). All but one of the hospitalized residents were children (6–15 years of age) (*9*).

Before onset of symptoms, 4 children from 1 household, 3 of whom had confirmed cases (including the index patient), were reported to have had close contact with the dead bodies of sick chickens (*15*). The 2 confirmed case-patients in the second household reportedly slaughtered a duck together on January 1, 2006, at the beginning of a die-off in the household’s flock (*14*). Two of the remaining confirmed case-patients lived in the third household and had no history of contact with sick or dying poultry. The remaining confirmed case occurred in a fourth residence located near the first household (*10*), but because we lacked information on the number of household members and the case-patient’s exposure history, we excluded it from these analyses. Most, if not all, of the 21 residents attended a dinner hosted by the family of the index patient on December 24, 2006, while he was symptomatic (*8*).

### Statistical Methods

We used a previously developed statistical transmission model (*16*,*17*) to test whether human-to-human transmission occurred, and if it did, to estimate transmission parameters. In the model, persons mix with one another in households and between households. In addition, we include a common source of infection due to zoonotic exposure. Mathematical and statistical details are given in the Technical Appendix.

#### Model of Probability of Transmission

We define *p*_1_ as the probability that an infectious household member infects another household member in 1 day. If the distribution of the infectious period is known, we can obtain the household secondary attack rate (SAR_1_) from *p*_1_, defined as the probability that an infectious household member infects another household member over his or her infectious period. Similarly, we define the daily transmission probability (*p*_2_) and the community SAR (SAR_2_) for between household spread. Finally, we define the daily probability (*b*) that any person is infected from a zoonotic source. The contact structure used for parameter estimation is shown in the Figure. We assume that the distributions of the incubation and infectious periods are predetermined by the investigator.

**Figure:**
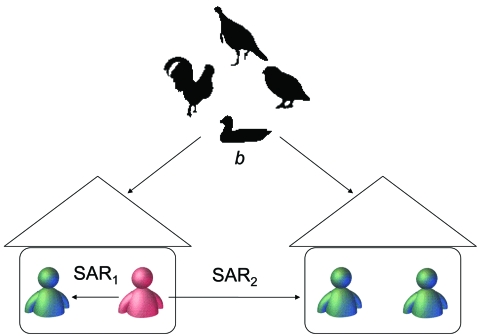
Schematic of estimation method. An infectious person (in red) infects a susceptible person (in green) in the same household with probability of household secondary attack rate (SAR_1_) and infects a susceptible person in a different household with probability SAR_2_. The common infectious source (i.e., avian hosts) infects a susceptible person with probability *b* per day. The likelihood function is constructed from symptom-onset dates and exposure information to estimate the above parameters

We establish the likelihood function for each person and then for the whole population for statistical inference. The likelihood function for a person is equivalent to the probability of observing the realized data on that person throughout the outbreak. The likelihood function for a person labeled *i* is built with the following steps: 1) Obtain the probability that person *i* is infected by an infectious source labeled *j* on day *t*, given person *i* is not infected up to day *t* – 1. If source *j* is a person, this probability is *p*_1_, for the same household, or *p*_2_ for exposure in the community, multiplied by the probability of person *j* being infectious on day *t*. The probability of person *j* being infectious on day *t* is derived from the symptom-onset day of person *j* and the distribution of the infectious period. If source *j* is zoonotic, the infection probability is *b*. The probability of escaping infection is simply 1 minus the corresponding probability of infection. 2) Take the product of the probabilities obtained in step 1 over all humans and zoonotic sources *j* to obtain the probability of person *i* escaping infection by any infectious source on day *t*. 3) Take the product of the probabilities obtained in step 2 over all days before and including day *t* to obtain the probability of person *i* escaping infection up to day *t*. 4) If person *i* is not infected by the end of the outbreak, the likelihood function for person *i* is the product of the probabilities of person *i* escaping infection up to the last day of observation. 5) If person *i* is observed to have symptom onset on day 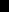
and the infection time is known to be *t*, the probability of the data regarding person *i* is the product of 3 pieces of information: a) the probability of person *i* escaping infection up to day *t –* 1, b) the probability that person *i* is infected on day *t,* and c) the probability that the duration of the incubation period is 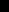
– *t*. Because we do not observe the infection time, the likelihood function for person *i* is obtained by summing the above product, a – c, over all potential values of *t*.

The likelihood function for the whole population is the product of all the individual likelihood functions. In the event that human-to-human transmission occurs, SAR estimates are used to estimate the local basic reproductive number (*R_0_*), which is defined as the average number of secondary cases infected by a typical index case-patient in the beginning of the outbreak (Technical Appendix). There is potential for sustained transmission if *R_0_* is >1. If human-to-human transmission is determined to be occurring, then the above parameters are estimated from the symptom dates and contact information from the population under study. Data on exposed persons who do not become ill form an important component of the inference procedure.

#### Statistical Test

We set up a statistical test with the null hypothesis being that no human-to-human transmission occurs, that is, *p*_1_ = *p*_2_ = 0. The alternative hypothesis is either *p*_1_ or *p*_2_ is not equal to 0, or both are not equal to zero. The test statistic we use is proportional to the ratio of the maximum value of the likelihood function assuming the null hypothesis is true (null likelihood) and the maximum value of the likelihood function at the estimated parameter values (full likelihood).

Specifically, we define the likelihood ratio test statistic as –2 log (the null likelihood function divided by the full likelihood function). If no human-to-human transmission occurs, the 2 likelihood functions would be roughly equal, and we expect to see a likelihood ratio close to 1, and, thus, a likelihood ratio statistic close to 0. A large value of the likelihood ratio statistic is evidence of deviation from the null hypothesis. The question is how to obtain a reference set of the likelihood ratio statistic values that we would see under the null hypothesis. Given no human-to-human transmission, all the observed case-patients must have been infected by the zoonotic source. Since the exposure to the zoonotic source is assumed constant for each person on each day, the null likelihood function will not change if we reassign the infection and symptom status of the observed case-patients to a different group of people in the population. By performing such reassignment many times, we obtained a collection of datasets that were each equally likely to have been observed had there been no human-to-human transmission. The values of the likelihood ratio statistic calculated from these datasets form the null distribution for statistical testing. This method is referred to as a permutation test. The p value is given by the proportion of the reference values that are equal to or larger than the observed likelihood ratio statistic value. More technical details are given in the online appendix.

The probability of infection by the zoonotic source may not be estimable together with SAR_1_ or SAR_2_ from an observed cluster. In such a situation, a statistical test of the occurrence of human-to-human transmission is still meaningful because the likelihood ratio test statistic is still estimable from the permuted datasets.

#### Data Required

A list of the inputs that are required for estimation and statistical testing are listed in the Table. Three categories of input parameters are required for this estimation model: outbreak-wide, individual level, and analysis parameters. The duration of the outbreak, the duration of the incubation period for the pathogen, and the minimum and maximum durations of the infectious period for the pathogen are the required outbreak-wide inputs. For each person, their residential location (neighborhood and household), their demographic characteristics (sex and age), and whether they were a case-patient or not are required input parameters. Case-patients require additional input of their illness-onset dates, types of outcome, outcome dates, and whether or not they are the index patient in the outbreak. Hospitalization and treatment dates (considered prophylactic for nonpatients) are optional input parameters for each person. For each person who visits another residence during the outbreak period, his or her identifiers, the neighborhood and household visited, and the start and end dates of the visit are required inputs. Analysis-related inputs include the last date of community exposure to potential common sources of infection, the last date of observation, and inputs for *R_0_* estimation (mean number of residents per household and mean number of out-of-residence contacts per person per day). An expanded version of the model will require the input of other exposure information such as from schools or hospitals.

**Table:** Parameters and data used in analysis

Category	Parameter/data	Required*
Entire outbreak	Outbreak begin date	X
	Outbreak end date	X
	Latent/incubation period, d†	X
	Infectious period, d†	X
All persons	Neighborhood of residence	X
	Household of residence	X
	Sex	X
	Age, y	X
	Case status (yes or no)	X
Case-patients	Whether outbreak index case-patient (yes or no)	X
	Date of illness onset	X
	Outcome (recovered, died, or don’t know/still ill)	X
	Date of outcome	X
	Dates of hospitalization	O
	Period of receiving treatment (dates)	O
Non–case-patients	Dates of hospitalization	O
	Period of prophylactic treatment (dates)	O
Inter-residence visits	Identifier for visiting person	X
	Neighborhood visited	X
	Household visited	X
	Dates of the visit	X
Analysis parameters	End of exposure to the common source of infection (date)	X
	Final day of observation (date)	X
R_0_ estimation	Mean no. residents per household	X‡
	Mean no. community contacts per person/d	X‡

*X, required; O, optional; R_0_, basic reproduction number. †The user defines the distribution of this period, including the minimum and maximum length of the period. ‡Required to estimate R_0_.

## Results

For the outbreak in Indonesia, Figure shows that the incubation period had a probable range of 3–7 days and the infectious period, a probable range of 5–13 days. Thus, we let the incubation period have a uniform distribution of 3–7 days (mean 5 days) and the infectious period a uniform distribution of 5–13 days (mean 9 days). For the data shown in Figure, only the household SAR (SAR_1_) can be estimated. We determined that human-to-human spread did occur by rejecting the null hypothesis of no human-to-human transmission (p = 0.009). The estimated household SAR is 0.29 (95% confidence interval [CI] 0.15–0.51). Thus, a single infected person in a household infected another household member with the probability of 0.29. The average household size for rural Indonesia is ≈5 people. Because we do not have an estimate of the community SAR, we have an estimate of the lower limit of the local *R_0_*,*_,_* i.e., 1.14 with a 95% CI of 0.61–2.14. A sensitivity analysis on the distribution of the incubation and infectious period shows that the test and estimates for SAR_1_ and *R_0_* are insensitive to uncertainty about these distributions within plausible ranges.

For the outbreak in Turkey, all the parameters are estimable, but we do not reject the null hypothesis of no human-to-human transmission (p = 0.114). Our estimate of the daily probability of infection from the common source is 0.011 (95% CI 0.005–0.025).

## Discussion

We have presented statistical evidence that the strain of HPAI (H5N1) that caused the family cluster of human cases in northern Sumatra was spread from human to human and that the household SAR was 29%. This household SAR is similar to statistical estimates for interpandemic influenza A in the United States (12.7%–30.6%) (*18*,*19*). The mean incubation period of this strain appears to have been ≈5 days, nearly twice as long as for past pandemic strains and current interpandemic strains of influenza. The CI for the estimated lower bound for the local *R_0_* covers 1. Therefore, even though we determined that human-to-human transmission probably occurred, whether the virus was capable of sustained human-to-human transmission is not clear. This virus may have required very close human contact to be transmitted. Even with no intervention, the finding that *R_0_* = 1.14 indicates that the chance that a single introduction would result in any further spread is ≈12%. In addition, the reported prophylactic use of oseltamivir may have played some role in limiting further spread. We did not find statistical evidence of human-to-human spread for the outbreak in eastern Turkey. This does not mean that no low-level human-to-human spread occurred in this outbreak, only that we lack statistical evidence of such spread. The power would be too low to detect such spread for an outbreak with 7 total cases and small SARs (*17*).

We did not consider the role of heterogeneity—such as age, sex, treatment status, or quarantine—in transmission. The parameters could be made to be functions of time-dependent covariates, as we have done with similar models (*16*,*19*,*20*). We can easily extend the model used here for covariates; however, we must have sufficient data to support such models.

Computer simulations have shown that the targeted use of influenza antiviral agents could be effective in containing a potential pandemic strain of influenza at the source (*21*,*22*), if initiated within 3 weeks of the initial case in the community, and if the *R_0_* is <1.8. This strategy, known as targeted antiviral prophylaxis, involves treating identified index patients in a mixing group and offering a single course of prophylaxis to the contacts of these index patients in predefined close contact groups, i.e., households at a minimum but also possibly neighborhood clusters, preschool groups, schools, and workplaces. In addition, the voluntary household quarantine of suspected close contacts of case-patients was recommended. Targeted antiviral prophylaxis at the household and neighborhood cluster level was carried out for the outbreak in Sumatra.

Ascertaining whether a potential pandemic strain of influenza is capable of sustained human-to-human transmission and estimating key transmission parameters are important. To estimate more than the household SAR, more detailed community data need to be collected. This would include a complete census of potentially exposed households and persons in the area where immediate transmission could occur from both potential zoonotic and human sources. Such data would enable estimation of important parameters and a more complete estimate of the *R_0_* rather than just the lower limit.

We have developed a software application, TRANSTAT, for implementing these analyses. This application provides a stand-alone environment for the entry, storage, and analysis of data from outbreaks of acute infectious diseases. A partial list of the input information is given in the Table. The statistical methods presented here can be applied to the data along with several standard epidemiologic tools. This information system would allow for real-time analysis and evaluation of control measures for an outbreak. We would encourage outbreak investigators to use this tool, taking care to input data on the exposed nonpatients as well as case-patients. The authors will provide a link to this software upon request.

## Supplementary Material

Technical Appendix Detecting Human-to-Human Transmission of Avian Influenza A (H5N1)

Appendix Figure 1 Exposure and disease events for each member of the family cluster in northern Sumatra, Indonesia. Dark boxes, duration of illness; white boxes without text, recovery period; thick dark vertical line, death; dark triangles, known contacts between members; shaded triangles, suspected contacts. *Unknown location of residence.

Appendix Figure 2 Exposure and disease events for each member of the family cluster in Eastern Turkey. Dark boxes, period of illness; white boxes without text, recovery period; thick dark vertical line, death; *Exposed to corpses of potentially diseased poultry. **Most of the members of houses 2 and 3 attended.
