# Supplementary material for: Detecting Human-to-Human Transmission of Avian Influenza A (H5N1)
Source: Emerg Infect Dis. 2007 Sep;13(9):1348–53. doi: 10.3201/eid1309.07-0111 (PMC2857285; doi:10.3201/eid1309.07-0111)
Supplement: Technical Appendix — Detecting Human-to-Human Transmission of Avian Influenza A (H5N1) [file 07-0111_Techapp-s1.pdf]

Technical Appendix

Detecting Human-to-Human Transmission of Avian Influenza A (H5N1)

The Likelihood

The Likelihood in a Prospective Design

We model the within household spread by the household transmission probability (p_1), defined as the probability that an infectious household member infects another household member in a 1 day period. The between household transmission probability (p_2) is the probability that an infectious person infects a person from a household different from his or her own in a 1 day period. Finally, b is the probability that any person is infected from a zoonotic source due to 1 day of exposure. By prospective design we mean that a population of size N are free of the disease at the beginning and followed up from day 1 to some day T . We assume the whole population is exposed to a constant zoonotic infective source throughout the observation period. Let $\tilde{t}_i$ be the symptom onset day for an infected person i . People who do not show any symptom by day T will have $\tilde{t}_i = \infty$ by default. Let $c_{ij}(t)$ indicate whether there is a close contact (1) or a casual contact (0) between persons i and j . The probability that an infective person j infects a susceptible person i on day t , is given by

$$p_{ji}(t) = p_1^{c_{ij}(t)} p_2^{1-c_{ij}(t)} f(t - \tilde{t}_j) ,$$

where $f(l)$ is the distribution of the infectious period. The probability that a susceptible person i escapes infection from all infective sources on day t is then given by

$$e_i(t) = (1-b)\prod_{j=1}^{N} p_{ji}(t) .$$

Assume that the duration of the latent period δ has the distribution $g(l) = \Pr(\delta = l)$, $l = \delta_{\min}, \delta_{\min}+1, \cdots, \delta_{\max}$. Define $\tilde{t} = \{\tilde{t}_j,\ j = 1, \cdots, N\}$, we construct the likelihood for person i as

$$L_i(b, p_1, p_2 \mid \tilde{t}) = \begin{cases} \prod_{t=1}^{T} e_i(t), & \text{Not infected.} \\ \sum_t g(\tilde{t}_i - t)(1 - e_i(t)) \prod_{\tau=1}^{t-1} e_i(\tau), & \text{Otherwise.} \end{cases}$$

The Likelihood in Case-Ascertained Design

Real epidemic data, such as the avian A(H5N1) influenza clusters discussed in this paper, generally come with the case-ascertained design instead of the prospective design, *i.e.*, the whole cluster is ascertained upon the development of disease (*i.e.*, symptom onset) of one or more index cases. To reduce the selection bias, the likelihood should be conditioning on the symptom status on the day when the index cases are ascertained. Let $\underline{t}_i = \tilde{t}_i - \delta_{\max}$ and $\overline{t}_i = \tilde{t}_i - \delta_{\min}$ be the earliest and latest possible infection days of person i, respectively. Let d_i denote the index case in the household of person i. Following Yang et al. (*1*), the marginal probability of having symptom onset later than $\tilde{t}_{d_i}$ is

$$L_i^m(b, p_1, p_2 \mid \tilde{t}) = \sum_{t=\underline{t}_{d_i}+1}^{\overline{t}_{d_i}} \{ (\prod_{\tau=\underline{t}_{d_i}+1}^{t-1} e_i(\tau))(1 - e_i(t)) \Pr(\tilde{t}_i > \tilde{t}_{d_i} \mid t)\} + \prod_{t=\underline{t}_{d_i}+1}^{\overline{t}_{d_i}} e_i(t) .$$

The joint conditional likelihood $L^c(b, p_1, p_2 \mid \tilde{t}) = \prod_i \frac{L_i(b, p_1, p_2 \mid \tilde{t})}{L_i^m(b, p_1, p_2 \mid \tilde{t})}$ will be maximized to obtain the maximum likelihood estimates. By conditioning, index cases do not contribute to the joint conditional likelihood.

The Permutation Test

The Hypotheses

Testing the existence of human-to-human transmission is equivalent to testing the hypotheses

$$\mathrm{H}_0 : p_1 = p_2 = 0 \quad vs.$$
$$\mathrm{H}_1 : p_1 > 0 \; or \; p_2 > 0,$$

where H_0 is the null hypothesis and H_1 is the alternative hypothesis.

The Test Statistic

When there is no person-to-person transmission, i.e., $p_1 = p_2 = 0$, expression (1) reduces to

$$e_i(t) = (1 - b).$$

Let $L_0^c(b \mid \tilde{t})$ denote the likelihood for the null model. The test statistic is defined as the likelihood ratio statistic:

$$\lambda = -2 \log \frac{\sup_b L_0^c(b \mid \tilde{t})}{\sup_{b, p_1, p_2} L^c(b, p_1, p_2 \mid \tilde{t})}.$$

The Null Distribution Based on Resampling

Yang et al. (*2*) discussed the permutation test for the prospective design, but the same logic applies to the case-ascertained design as well. When the observed data are truly generated from H_0, *i.e.*, there is no human-to-human transmission, we can reassign all of the observed symptom onset days (and associated infection status) to a different subset of people, and every such rearrangement has the same likelihood L_0^c. By permuting symptom onset days across the population, we obtain an empirical distribution of the test statistic which can then be used to approximate the null distribution of the test statistic under H_0. Let $(\tilde{t}_1^{[k]}, \tilde{t}_2^{[k]}, \cdots, \tilde{t}_N^{[k]})$ be the kth permuted sample of $(\tilde{t}_1, \tilde{t}_2, \cdots, \tilde{t}_N)$, and $\lambda^{[k]}$ be the corresponding test statistic, $k = 1, \cdots, M$. Then the p-value is given by $\frac{1}{M} \sum_k I(\lambda \geq \lambda^{[k]})$. This permutation test can be refined by varying symptom onset days of infected individuals in any given permuted data, as long as the null likelihood L_0^c is unchanged. The refined permutation test resamples data from a much larger sampling space and thus can attain higher statistical power. Technical details of these resampling methods can be found in Yang et al. (*2*). However, an important distinction from Yang et al. (*2*) is that the index cases should not be involved in the refinement stage for the case-ascertained design, because changing the symptom onset days of the index cases also changes the index or non-index status and makes it difficult to keep L_0^c constant.

Three Important Functions of Estimated Parameters

We summarize the degree of transmissibility of the infectious agent by the household secondary attack rate (SAR_1), where $SAR_1 = \sum_l f(l)\left(1-(1-p_1)^l\right)$, and the community secondary attack rate, (SAR_2), where $SAR_2 = \sum_l f(l)\left(1-(1-p_2)^l\right)$.

The basic reproductive number (R_0) is the average number of people that a typical infected person infects in an entirely susceptible population. For a community of size N with average household size M, we have the local $R_0 = (M-1) \times SAR_1 + (N-M) \times SAR_2$. The maximum likelihood estimates of SAR_1 and SAR_2 are used to get an estimate of R_0. If only an estimate of SAR_1 is available, then a lower bound on R_0 can be estimated.

References

1. Yang Y, Longini IM, Halloran ME. Design and evaluation of prophylactic interventions using infectious disease incidence data from close contact groups. Appl Statist. 2006;55:317–30.

2. Yang Y, Longini IM, Halloran ME. A resampling-based test to detect person-to-person transmission of infectious disease. Annals of Applied Statistics. 2007;1:211–28. Available from http://projecteuclid.org/dpubs/repository/1.0/disseminate?handle=euclid.aoas/1183143736&view=body&content-type=pdfview_1
