# Supplementary figures and images for: Detecting Human-to-Human Transmission of Avian Influenza A (H5N1)
Source: Emerg Infect Dis. 2007 Sep;13(9):1348–53. doi: 10.3201/eid1309.07-0111 (PMC2857285; doi:10.3201/eid1309.07-0111)

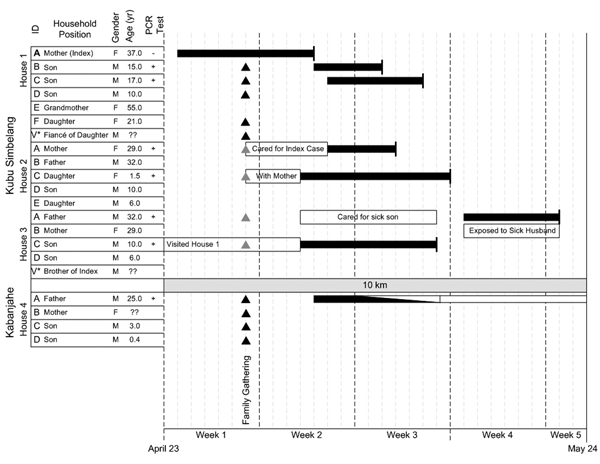

Supplement: Appendix Figure 1 — Exposure and disease events for each member of the family cluster in northern Sumatra, Indonesia. Dark boxes, duration of illness; white boxes without text, recovery period; thick dark vertical line, death; dark triangles, known contacts between members; shaded triangles, suspected contacts. *Unknown location of residence. [file 07-0111_appF1-s2.gif]

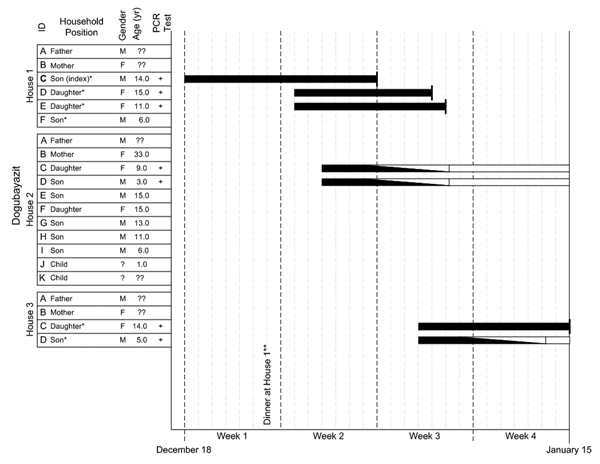

Supplement: Appendix Figure 2 — Exposure and disease events for each member of the family cluster in Eastern Turkey. Dark boxes, period of illness; white boxes without text, recovery period; thick dark vertical line, death; *Exposed to corpses of potentially diseased poultry. **Most of the members of houses 2 and 3 attended. [file 07-0111_appF2-s3.gif]
